# Telling Lies: The Irrepressible Truth?

**DOI:** 10.1371/journal.pone.0060713

**Published:** 2013-04-03

**Authors:** Emma J. Williams, Lewis A. Bott, John Patrick, Michael B. Lewis

**Affiliations:** School of Psychology, Cardiff University, Cardiff, United Kingdom; University of Leicester, United Kingdom

## Abstract

Telling a lie takes longer than telling the truth but precisely why remains uncertain. We investigated two processes suggested to increase response times, namely the decision to lie and the construction of a lie response. In Experiments 1 and 2, participants were directed or chose whether to lie or tell the truth. A colored square was presented and participants had to name either the true color of the square or lie about it by claiming it was a different color. In both experiments we found that there was a greater difference between lying and telling the truth when participants were directed to lie compared to when they chose to lie. In Experiments 3 and 4, we compared response times when participants had only one possible lie option to a choice of two or three possible options. There was a greater lying latency effect when questions involved more than one possible lie response. Experiment 5 examined response choice mechanisms through the manipulation of lie plausibility. Overall, results demonstrate several distinct mechanisms that contribute to additional processing requirements when individuals tell a lie.

## Introduction

People lie surprisingly often, a task which requires a number of complex processes [1]. For example, 40% of adults have reported telling a lie at least once per day [2]. The majority of these lies are likely to be trivial in nature, serving a communicative function [3]–[5], however, others can have more drastic consequences, such as those told by criminal witnesses and suspects [6]–[10]. Despite the apparent prevalence of lie-telling within society, lying is a complicated behavior that requires breaking the normal, default rules of communication [11]. The liar must first of all decide not to assert the truth, and then must assert an alternative statement that is plausible and appears informative to the listener, all the while concealing any outward signs of nervousness. Such a pragmatic feat requires cognitive processes in addition to those used when telling the truth. In this article we investigate what those processes might be. As such, we are less interested in the intent to instil a false belief in another’s mind but more interested in the necessary and universal cognitive processes associated with making a statement that is not true. The research presented here may be far removed from an aggressive interrogation where lives or liberty are at stake; but, the fundamental cognitive processes that are taking place when someone either tells the truth or constructs a falsehood are going to have some aspects in common regardless of the situation. The aim of the current research is to understand better these cognitive processes.

Our starting point is to examine the reasons given in the literature for why lying appears to be more difficult than telling the truth. Longer lie times, for example, must be indicative of additional cognitive processes involved in lying compared to telling the truth. Based on a framework developed in 2003 [1], we will discuss three processes that have been implicated in lying and summarise the empirical evidence in favour of each.

### Suppression of the truth

Our default communicative stance is to tell the truth. Without the assumption that speakers utter the truth most of the time, it is difficult to see how efficient communication could ever occur [11]. This suggests that when people wish to lie to a question they will need to intentionally suppress the default, truthful response, which should increase the difficulty of lying relative to telling the truth.

There is indeed plenty of empirical evidence consistent with the claim that telling lies involves suppressing the truth. For example many researchers have found longer response times for lying relative to telling the truth [1], [12]–[17], and there is neuroscientific evidence that brain regions active in lying overlap with brain regions associated with general response inhibition [18]–[22].

A number of these studies have been based around a lie detection technique known as the Concealed Information Test (CIT) [23]. This typically involves the presentation of a variety of different images or words via a computer screen. Some of these stimuli relate to previously learned information, known as probes, whereas others are irrelevant items. In practical situations, individuals may be asked the identity of a murder weapon, with the probe item being an image of the actual murder weapon (i.e., a knife) embedded within a series of irrelevant images (i.e., a gun, a hammer, a baseball bat). Participants are instructed to deny recognition of all items. If participants have concealed knowledge and recognise the murder weapon, they are expected to respond differentially to probe and irrelevant items. Although traditionally used to examine physiological responses, such as skin conductance [16] and event related potentials [24]–[26], this paradigm has recently been used with response times to successfully discriminate “guilty” from “innocent” participants, with guilty participants taking longer to deny recognition of probes than irrelevant items [16], [27], [28]. It has been argued, however, that such paradigms measure the possession of concealed knowledge rather than deception per se [14] and therefore may not allow us to fully elucidate the distinct processes involved in responding to questions deceptively.

These findings have meant that recent cognitive models of deception have incorporated both the automatic activation of the truth and its resultant suppression as additional processes that contribute to longer response times for liars [1], [20], [29]–[31]. For example, the Activation-Decision-Construction Model (ADCM) [1], [31] claims that following a question, relevant information (in particular, the truth) is automatically activated in long-term memory [32]. This information is then made consciously available in working memory [33]. In order to respond to a question deceptively, cognitive resources are required to inhibit the truthful response. Similarly, the Working Model of Deception (WMD) [30] highlights response inhibition as a pre-requisite to responding to a question deceptively.

While the need to suppress the truth is undeniably an important component of why lying is more difficult than telling the truth, there are several other reasons that have received less attention in the literature and that might also contribute. These are discussed below.

### The decision to lie

Assuming that people tell the truth by default [11], they must make a conscious choice to lie. The decision to lie is therefore likely to be an additional cognitive process associated with lying that takes time to execute. Indeed, current models of how we lie include a lie decision component. For example, the Working Model of Deception (WMD) [30] assumes that when an individual hears a question to which they may respond deceptively, executive control processes are used to determine the appropriate response (i.e., lie or truth), with a decision being made based on the likely risks and benefits involved. Similarly, the Activation Decision Construction Model Revised (ADCM-R) [34] considers individuals who have previously decided to lie to particular questions and have rehearsed an answer. In these cases, the model states that a decision is still required because individuals must remind themselves of their decision to lie when that particular question is heard.

Despite the inclusion of decision components in the models, there is surprisingly little work that has specifically investigated how people make the decision to lie. This is perhaps because it is experimentally much easier to instruct people when to lie than to allow them to choose. We can find only a few papers that have investigated the decision process [21], [31]. The first of these [31] presented participants with a selection of neutral questions and questions probing embarrassing information. Participants were instructed to lie to certain questions, such as those regarding their employment history, and tell the truth to others, such as those regarding what they did on Sunday morning. However, for general questions, they were instructed to answer truthfully unless a question probed embarrassing information about which they would normally lie to a stranger, in which case they should lie. In this condition, participants needed to decide themselves when to lie and when to tell the truth. The experiment demonstrated that more time was needed to respond when individuals chose to lie compared with when they had been instructed, and both took longer than telling the truth, consistent with the idea that the decision of how to respond adds to cognitive processing load. However, it is difficult to be certain whether the elevated response times were due to the evaluation of whether a question was embarrassing or to the decision of how to respond.

The second of these papers [21] allowed participants to choose whether to lie or tell the truth to computer-generated yes-no questions regarding an embarrassing past life event, although participants were asked to achieve an approximate balance between truths and lies over the course of the experiment. Brain activity (using neuroimaging techniques) was recorded rather than behavioural data. Similar to findings when individuals have been instructed on how to respond [12], [15], [20], [35], lying showed increased activation of the ventrolateral prefrontal cortices (implicated in deceptive capabilities [20]) compared to truth-telling. However, because there was no direct comparison of trials between choosing to respond and being instructed to respond, little can be concluded about the decision process itself.

### Construction of the lie

Lies and truths also differ in the way in which they are constructed. It is often the case that more than one possible lie is available. In this case the particular lie produced needs to be explicitly chosen from a range of alternatives. For a lie to be convincing then it must be plausible and consistent with previous information and so selecting such a lie introduces additional constraints. Truths, on the other hand, seem to be generated automatically without a need to always select “which” truth, since stimulus questions must merely be evaluated in relation to known information [36]. The procedures needed to choose which lie to use and to verify the plausibility may be costly to operate.

One study [31]directly tested whether the added complexity of lie construction was a contributing factor to elevated lie response times. Their approach was to manipulate whether participants responded to open-ended questions, such as, “What color is your hair?” or yes/no questions, such as, “Is your hair brown?”(Although we appreciate that differing definitions of open-ended questions exist, for clarity we use the same terms as the above cited paper). It was argued that more lie construction was needed to respond to open-ended questions than yes/no questions because open-ended questions required explicit retrieval of information from long-term memory, whereas yes/no questions merely needed the production of an affirmation or denial. If lie construction was contributing to longer lie response times, then lying to open-ended questions should be more difficult than lying to yes/no questions. Consistent with these predictions, longer lie response times were observed in the open-ended question condition than in the yes/no condition [31]. There are a number of issues that make the interpretation of this result difficult, however. First, while lying to open ended questions was slow relative to yes/no questions, telling the truth was also slow. It is therefore not clear whether their effect relates to lie construction or to the difficulty of responding to open-ended questions in general. Second, the content of the question was not equated across yes/no and open-ended conditions. For example, response times to questions such as “Do you like chocolate” were compared with questions such as “How many credit cards do you own?” Differences in response times could therefore be explained by differences in the ease of accessing information, rather than the question types *per se*.

While there has been no direct evidence about how people assess the plausibility of potential lies, there is indirect evidence that complex lies are costly to generate. If a person needs to monitor plausibility of a lie then this will be more difficult for more complex lies. First, studies investigating the effects of making lies more complex have found that they are easier to detect. For example, asking participants to recall events in reverse order [10] and using interview techniques that require longer answers to questions [37] have increased discrimination between liars and truth tellers. Finding that lies are easier to detect when the lie is more complex suggests that extra resources are needed to construct the plausible lie.

Secondly, if lie construction independently contributes to the processing difference between lying and truth-telling, individuals who have been given the opportunity to rehearse or prepare a lie response will require less processing time than unprepared liars. Several studies have found evidence that this is the case. A review of the literature conducted in 1981, found that the response time difference between lying and truth-telling only occurred when participants had not rehearsed a response [17]. A recent meta-analysis of 158 cues to deception similarly found that longer response times for liars only demonstrated a significant effect size when participants were not given the opportunity to prepare their lie [38]. Alternative paradigms incorporating an explicit period of rehearsal have shown smaller response time differences between rehearsed lies and truths compared to unrehearsed lies and truths [34].

In summary, we have reviewed the evidence for three processes involved in lying that are not involved in telling the truth. There is substantial evidence that the first process, the suppression of the truth, contributes to the extra costs involved in lying, but the evidence for the other processes is weaker. Our study therefore concentrates on testing whether the decision to lie and the construction of the lie contribute to the greater difficulty of lying, as distinct from suppressing the truth. In doing so, we hope to understand in more detail what cognitive processes are involved when people lie.

### Cognitive load

The aspects of lying described above all arguably add to the cognitive load of the process. Adding additional cognitive load to a deception situation has been shown to be effective in lie detection research. In support of this idea, studies investigating the effects of making lies more complex have found that they are easier to detect. For example, asking participants to recall an event in reverse order [10] or using interview techniques that require longer answers to questions [37], have been shown to increase discrimination ability between liars and truth tellers.

Although cognitive load provides a basis for current theoretical considerations of deception, its underlying mechanisms and processes are not fully understood. For example, while the cognitive load approach suggests that telling a lie is cognitively more complex than telling the truth and will result in behaviour that highlights this additional mental effort, such as a decrease in body movements and an increase in response time, there is no in-depth explanation of precisely why deception is more cognitively challenging, or the particular processes involved in any deceptive encounter. This is what the current study aims to explore.

## The Current Study

Our paradigm involved presenting participants with a colored square and asking them to lie or tell the truth about the color. We used vocal onset time as the dependent measure. This paradigm allowed us to focus on two main aspects of the lie process, namely, the suppression of factually truthful information and the production of an alternative, false response, since both should be required when falsely describing the color of a square. In Experiments 1 and 2 we investigated the decision to lie by comparing trials in which participants chose whether to lie or tell the truth compared to being instructed. In this way, it was possible to evaluate whether the process of making the decision to lie had a carryover effect into the lie itself. In the real world setting a person needs to decide to lie rather than being directed to lie and so in our pared-down version of the process it would be important to know whether differences present when deciding to lie are the same as those when directed to lie. In Experiments 3, 4 and 5 we investigated the lie construction process by comparing one possible lie response to a choice of two or three lie response possibilities, and by manipulating the plausibility of particular lie responses.

The color-naming paradigm that we have developed is different to the paradigms generally used in lie research. For example, in previous studies, participants have watched a simulated crime and lied about the protagonist [39], or been questioned by an interviewer regarding their background and instructed to lie about certain details [40]. The reason for the difference in methodology is that most of the previous research into lying has been concerned with lie *detection* whereas we are interested in the underlying cognitive processes. Deception researchers, understandably, are interested in the measure which is most able to distinguish lies from truths, whether that is skin conductance [41], facial expressions [42], or offline measures such as linguistic analyses [43], none of which are necessarily indicative of cognitive processes.

When researchers have used more traditional cognitive markers of deceit, such as response times, the emphasis has been on discovering whether a difference between lies and truths exists and how these compare to other ways of differentiation of deception [16]. Our experiments were designed to isolate the individual components of lying, however, which required eliminating as much variability as possible. We therefore removed factors such as the stress associated with lying, or the incentive to lie, which by their nature may variably affect the process [14], [15], [44], [38]. We consider the processes investigated here – the suppression of the truth and the production of alternatives – to be involved in every instance of lying and are therefore fundamental to the cognition of lying. Stress, the incentive to lie, and other situational factors need to be considered beyond the basic cognitive processes considered here.

## Experiment 1

There were two goals for Experiment 1. First, to establish whether our paradigm produced results consistent with the past literature on lying; specifically, that lie responses require slower response times than true responses [15], [45]. Second, we wanted to investigate the effects of deciding to lie by manipulating whether participants chose to lie, or whether they were directed to lie. Thus, prior to the presentation of the colored square, participants were either presented with an instruction to lie or tell the truth in their response or were given a choice between the two. On the latter trials, participants had to input their decision (lie or truth) on the keyboard. Once the square was presented, participants had to vocally respond with either the true color of the square, or lie about its color. We reasoned that the decision-making process would be involved in the former but not the latter condition and this would be reflected in differences in lying latency.

Different decision processes make different predictions about the interaction between the type of instruction (*directed* or given a *choice*) and the *honesty* of the response (*truth* or *lie*). We consider two possibilities. First, the decision to lie could be a departure from the normal, truth-telling state. Deciding to lie, rather than adhering to the default truth, would therefore require extra processing effort. This is the basic idea behind the decision components of the ADCM [34] and WMD [30]. If the decision to lie is more difficult than the decision to tell the truth, participants should need relatively longer to lie than to tell the truth in the choice condition compared to the directed condition. In short, there should be an interaction between instruction and honesty with a larger difference between lies and truths in the choice condition. Second, deciding to lie could be no different to deciding to tell the truth. As such, the having to make the decision will not impact upon the size of the lie/truth difference in reaction times. Having to choose a response would generally be more difficult than being directed on the response and so longer overall latencies might be expected for the directed compared to the choice conditions, and longer lie latencies than truth latencies. Under this account then, only main effects of type of instruction and honesty would be expected.

### Method

#### Participants

Twenty-one Cardiff University undergraduate psychology students volunteered for this study in exchange for course credit. Of these, 20 were female. Participants had a mean age of 19.52 (*SD*  =  0.68; *Range*  =  18–21) and spoke English as their first language. For this experiment, and all subsequent reported experiments, ethical approval was granted by the School of Psychology Ethics Committee at Cardiff University. In accordance with this, informed written and oral consent was obtained from all participants prior to the experimental task.

#### Design

A 2 x 2 within-subjects design was used, with the independent variables being honesty of response (lie vs. truth) and type of instruction (choice vs. directed). The dependent variable was response time. A total of 192 trials were included, with 64 from the directed to lie condition, 64 from the directed to tell the truth condition and 64 from the choice condition. The order of trials was randomised for each participant.

#### Procedure

**Figure 1:**
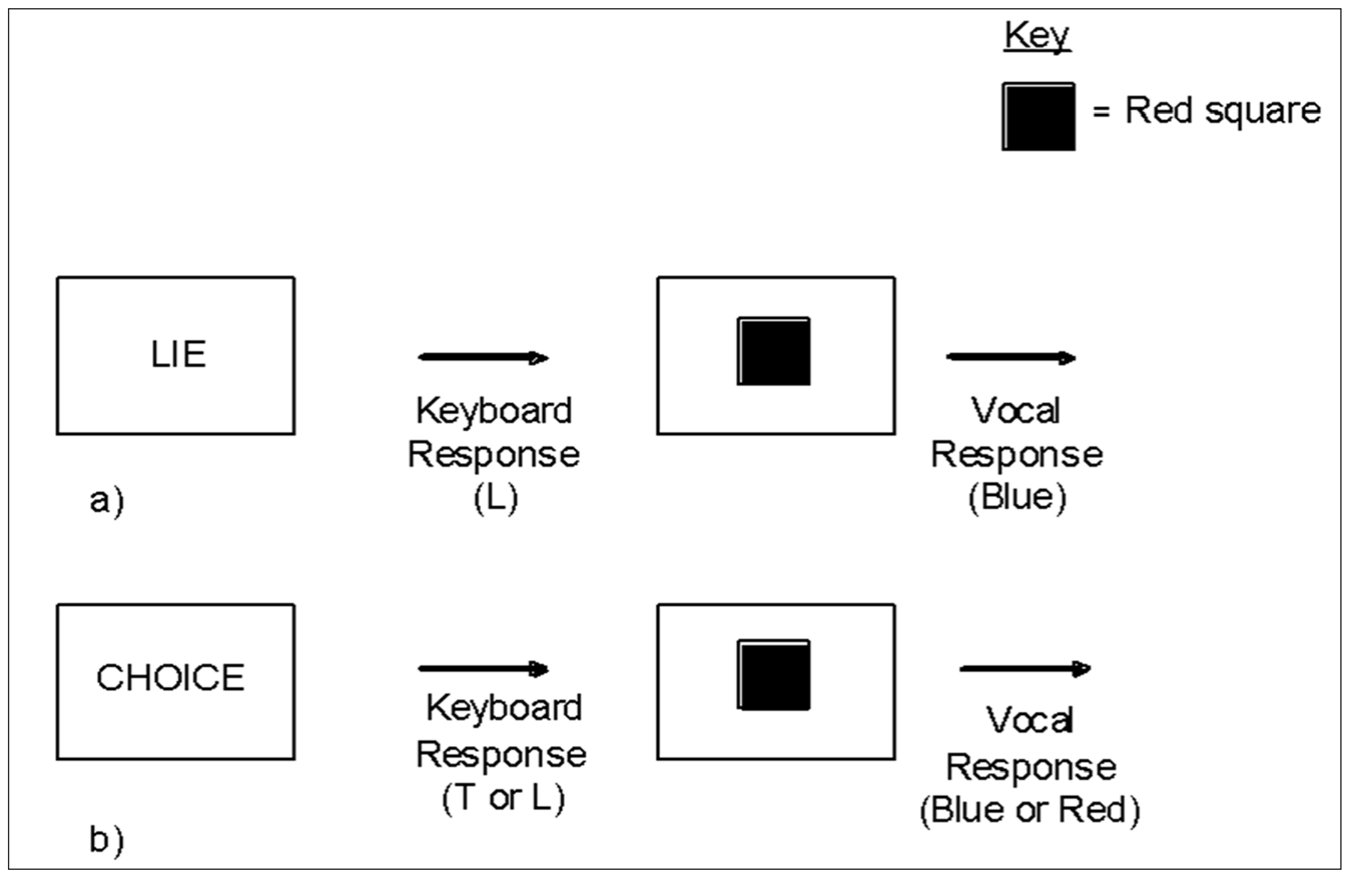
Example of trials in Experiment 1: a) Directed, b) Choice.

The experiment progressed as a series of trials each of which began with the presentation of one of three words in the centre of the computer screen (LIE, TRUTH or CHOICE). Participants were asked to indicate whether they understood by pressing the ‘T’ key when presented with the word ‘TRUTH’, the ‘L’ key when presented with the word ‘LIE’ and either the ‘T’ or ‘L’ key when presented with the word ‘CHOICE’, according to whether they chose to lie or tell the truth. Participants were asked to choose to lie and choose to tell the truth at least 10 times each, to enable data from both responses to be collected. The word remained on the screen until the participant pressed the appropriate button and was then replaced with either a blue or a red square. Participants then had to say either the true color of the square or lie about the color of the square by claiming that it was the opposite color (e.g., blue if it was red). Voice key responses were recorded via a clip microphone. An example of a directed trial and a choice trial are presented in Figure 1. After the vocal response was made, the next trial began after 500 ms. Instructions were presented on the screen and emphasised the importance of responding both as quickly and as accurately as possible. Participants took part in a practice block of 12 trials identical to the main trials. The question ‘What color is the square?’ was visually presented prior to both the practice block and the block of main trials. All stimuli were presented on a black background, with the squares being of equal size and the text being presented in Arial font, size 40.

### Results

Two subjects were removed from the analysis because they failed to follow experimental instructions of choosing to lie at least 10 times in the choice condition. All participants chose to tell to truth at least 10 times.

We treated response times greater than 2 s (approximately 3 SDs above the grand mean) as outliers in all of the experiments reported in this paper. Response times longer than this represented an excessively long time to retrieve the name of a color, and we found that using this cut-off meant that a similar number of outliers were eliminated across conditions. There were 103 (less than 3%) outliers in total, with 95 of these being a result of microphone problems (the microphone failed to pick up the initial answer). No responses were less than 100 ms. Inaccurate responses (132) were also removed from the analysis. Overall, there were 13 (2.0%) inaccurate responses in the choice lie condition and 53 (7.9%) in the choice truth condition, X^2^(1)  =  25.6, *p* < 0.05. There were 36 (2.7%) errors in the directed lie condition and 30 (2.2%) in the directed truth condition, X^2^(1)  =  0.6 *p* > 0.05. In total, 235 out of 3,648 data points were removed from the analysis.

**Figure 2:**
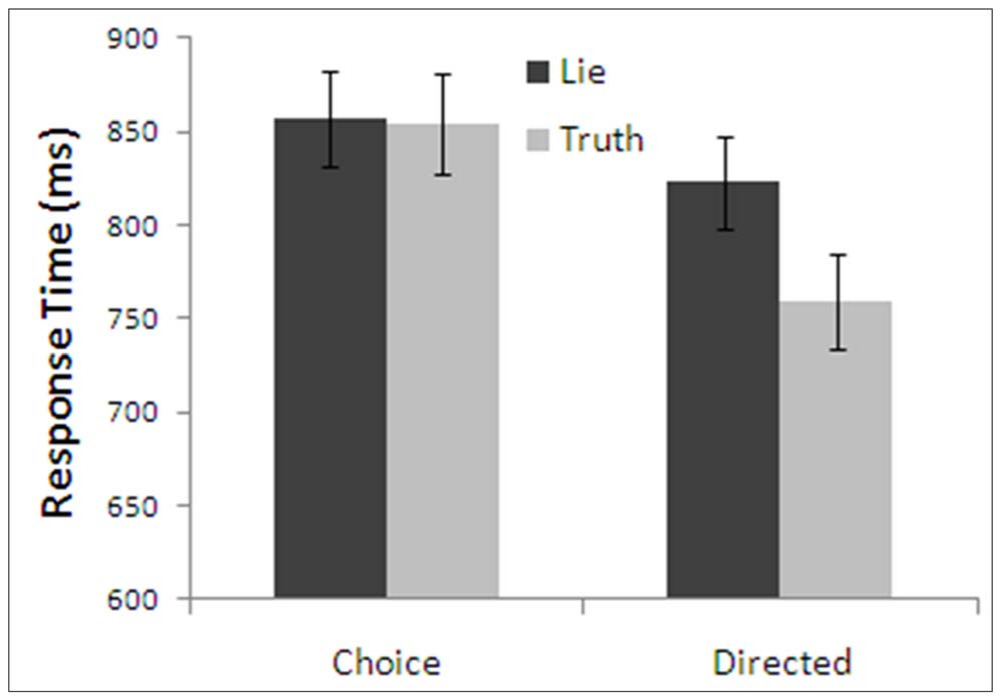
Response times of Experiment 1 as a function of type of instruction and honesty. Note: Error bars are standard error.

Mean response times for the four possible treatment combinations are presented in Figure 2. In contrast to either of the hypotheses considered above, there appears to be a large difference between truth and lies in the directed condition but not in the choice condition. To test this pattern we conducted a repeated-measures ANOVA with factors of type of instruction and honesty of response. We found a main effect of honesty with true responses being faster than lie responses, *F*(1,18)  =  7.89, *p* < .05, η^2^  =  .31, and a main effect of type of instruction with responses in the choice condition being longer than in the directed condition, *F*(1,18)  =  17.28, *p* < .001, η^2^  =  .49. The interaction was also significant, *F*(1,18)  =  9.97, *p* < .005, η^2^  =  .36. The faster production of true than lie statements was significant in the directed condition, (Directed - Truth: *M*  =  758.85, *SD*  =  111.08; Directed - Lie: *M*  =  822.98, *SD*  =  110.86; *F*(1,18)  =  21.88, *p* < .001, η^2^  =  .51), but not in the choice condition, (Choice - Truth: *M*  =  854.02, *SD*  =  118.12; Choice - Lie: *M*  =  857.39, *SD*  =  109.83; *F*(1,18)  =  0.40, *p*  =  .84, η^2^ < .01, *CI*  =  [–32, 38]).

### Discussion

When directed to lie or tell the truth, participants in our experiment needed on average 60 ms longer to lie than to tell the truth. This result demonstrates that our paradigm produces data consistent with previous research investigating response time and lying [1], [15], [31]. One way in which this result extends previous work, however, is that the role of the lie construction process was minimal in our experiment. Participants did not have to consider what an appropriate lie response might be (the only possible lie response was the alternate color) nor did they have to construct a convincing lie sentence. The most likely explanation for the differences in lie times is therefore that participants needed time to suppress the truth when lying.

The main aim of Experiment 1 was to investigate the effects of deciding to lie over being directed to lie. We were interested in whether there was a cost associated with deciding to lie in particular [34] or whether there was a general cost associated with having to choose a response compared to being directed. Surprisingly, the findings of Experiment 1 were not consistent with either of these possibilities. Although we observed an interaction between honesty of the response and the type of instruction, the difference between lying and telling the truth was significantly greater in the directed condition than in the choice condition; indeed, there was no significant difference between lying and telling the truth in the choice condition and there were significantly more errors in the truth condition. Before discussing the theoretical implications of these findings, however, we consider one factor that could have obscured differences between conditions in the choice condition.

Participants were slower to respond overall when they had to choose their response type than when they were directed on the response type. Also, participants were making more errors in the choice condition. In the choice condition, participants pressed a button to indicate their choice, whereas in the directed condition participants saw the word “truth” or “lie”. Participants therefore received a visual prompt regarding the response type in the directed condition but not in the choice condition. A greater degree of uncertainty about the expected response in the choice condition could therefore explain longer latencies overall, which could in turn have obscured honesty differences. We address these problems in Experiment 2 by providing a visual prompt to participants in both the choice condition and the directed condition.

## Experiment 2

Experiment 2 used a similar design to Experiment 1 except that participants were given a visual reminder of their decision in the choice condition, just as they were in the directed condition.

### Method

#### Participants

Twenty-three Cardiff University students were paid for participation in the experiment. Of these, 14 were female. Participants had a mean age of 21.65 (*SD*  =  4.59; *Range*  =  18–37) and spoke English as their first language.

#### Design

The design of the experiment was the same as that shown in Experiment 1. However, we increased the total number of trials to 200 to ensure an equal number in the choice and directed conditions overall (100 in the choice condition, 50 in the directed to lie condition and 50 in the directed to tell the truth condition).

#### Procedure

**Figure 3:**
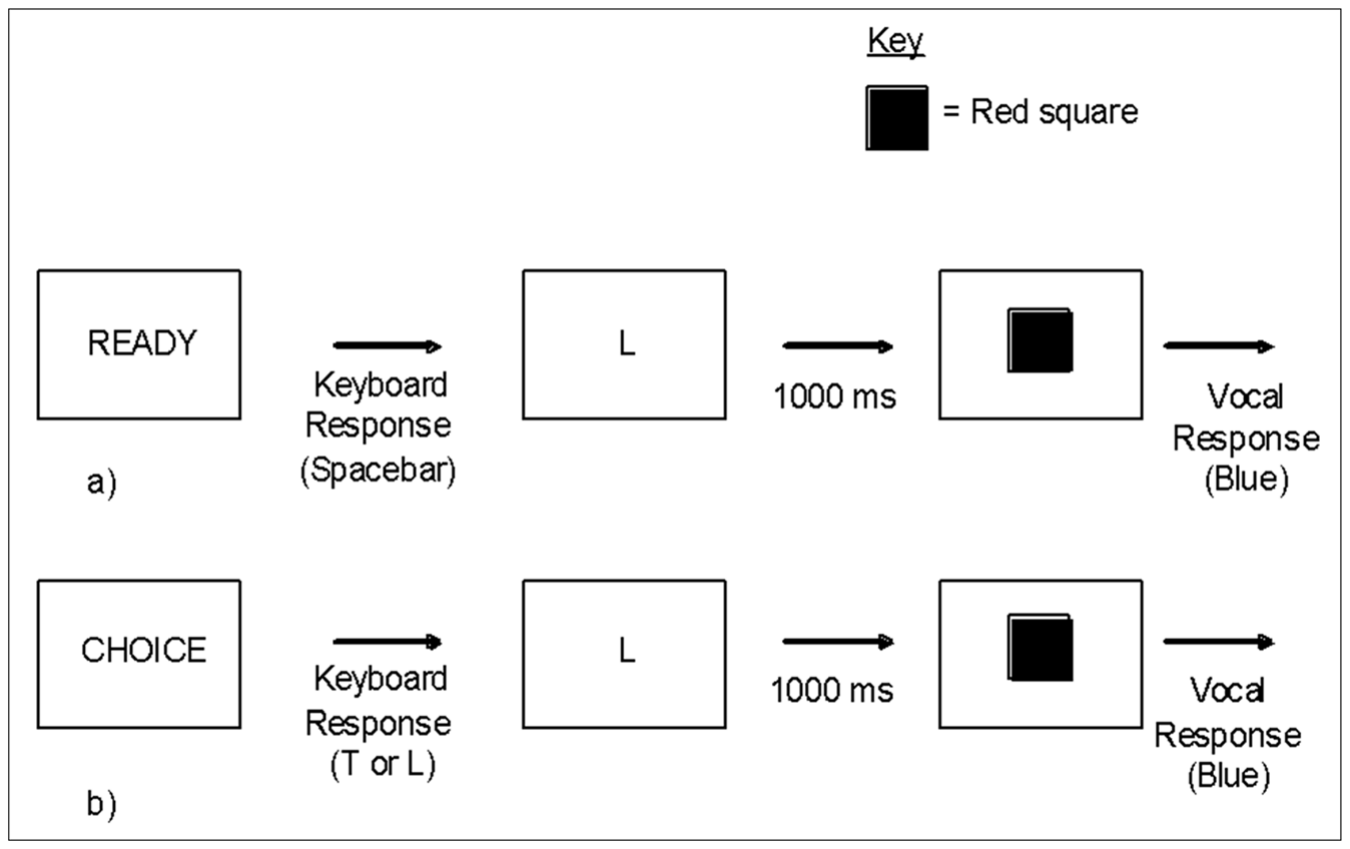
Example of trials in Experiment 2: a) Directed, b) Choice.

The task was a modified version of that described in Experiment 1 and involved the presentation of one of two words in the centre of the computer screen (READY or CHOICE). When the word ‘READY’ was presented, participants were instructed to press the space bar. When the word ‘CHOICE’ was presented, participants could press either the ‘T’ or the ‘L’ key, depending on whether they had chosen to tell the truth (T) or lie (L). On a ‘READY’ trial, the key press was followed by either the letter ‘L’ (relating to lie) or ‘T’ (relating to truth) presented in the centre of the screen for a one second period. On a ‘CHOICE’ trial, the key press was followed by a visual reminder of what key was pressed by presenting either an ‘L’ or a ‘T’ in the centre of the screen for a one second period. A colored square would then appear on the screen and the participant would report its true color or lie about it. The time taken to do this was recorded via a voice key. Examples of a directed and a choice trial are presented in Figure 3. The presentation of visual prompt was the only aspect of the procedure that differed from Experiment 1.

### Results

One participant was removed from the analysis because they failed to follow experimental instructions of choosing to lie at least 10 times, providing a final sample size of 22. There were 100 outliers (2.3%) in total, with 67 of these being a result of microphone problems. No responses were less than 100 ms. These were removed from the analysis. Inaccurate responses (126) were also removed from the analysis. There were 25 (2.3%) inaccurate responses in the choice lie condition and 53 (4.8%) in the choice truth condition, X^2^(1)  =  10.4 *p* < 0.05. There were 28 (2.5%) errors in the directed lie condition and 20 (1.8%) in the directed truth condition, X^2^(1)  =  1.4, *p* > 0.05. In total, 226 out of 4,400 data points were removed from the analysis.

**Figure 4:**
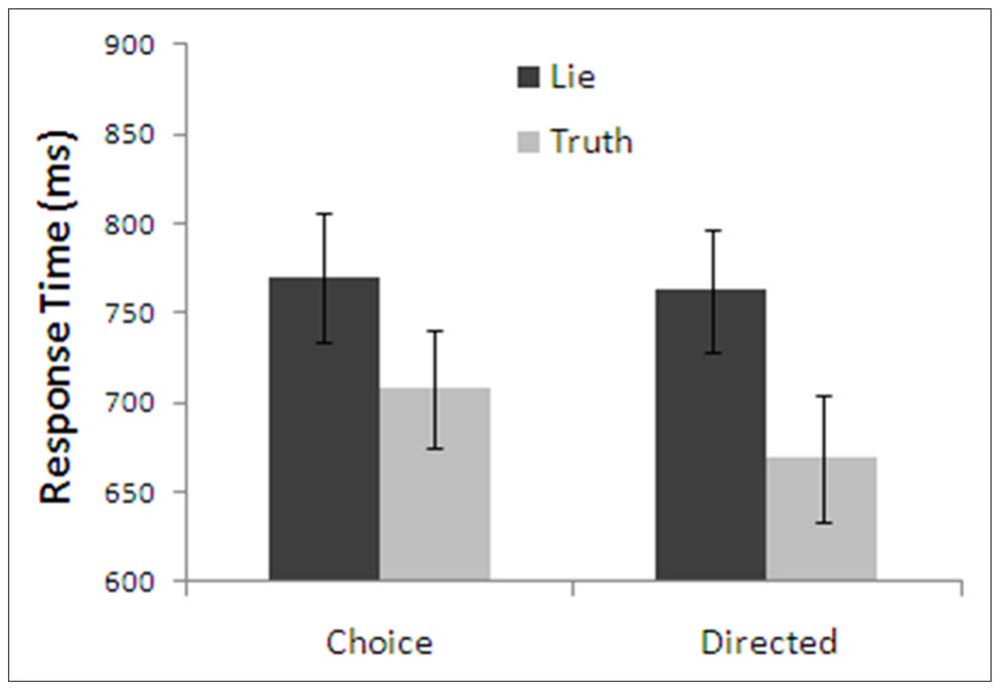
Response times of Experiment 2 as a function of type of instruction and honesty. Note: Error bars are standard error.

Mean response times for the four possible treatment combinations are presented in Figure 4. Overall, telling a lie took longer than telling the truth, *F*(1,21)  =  84.66, *p* < .001, η^2^  =  .80. Choosing how to respond took longer than being directed, *F*(1,21)  =  5.55, *p* < .05, η^2^  =  .21. There was also a significant interaction between the type of instruction and honesty of response, *F*(1,21)  =  5.93, *p* < .05, η^2^  =  .22, such that there was a greater difference between lying and telling the truth in the directed condition, (Directed - Truth: *M*  =  668.73, *SD*  =  142.87; Directed - Lie: *M*  =  763.06, *SD*  =  159.57), than in the choice condition, (Choice - Truth: *M*  =  707.83, *SD*  =  152.75; Choice - Lie: *M*  =  769.94, *SD*  =  167.12). This shows a similar pattern to Experiment 1, where a response time difference for lies and truths was only shown in the directed condition. Simple main effects analysis found that the effect of honesty of response was present in the directed condition, *F*(1,21)  =  80.30, *p* < .001, η^2^  =  .79 and, in contrast to Experiment 1, it was also present in the choice condition, *F*(1,21)  =  31.82, *p* < .001, η^2^  =  .60. Participants also took longer to respond when they chose to tell the truth compared to when they were directed to tell the truth, *F*(1,21)  =  16.65, *p* < .001, η^2^  =  .44, whereas there were no differences in response times when individuals chose to lie compared to when they were directed to lie, *F*(1,21)  =  0.25, *p*  =  .62, η^2^  =  .01, *CI*  =  [–21, 35].

### Discussion

The results of Experiment 2 provide further support for the finding that telling a lie takes significantly longer than telling the truth. In contrast to the findings of Experiment 1, this occurred both when individuals were directed in their response and when they chose their response. Furthermore, we no longer observed that responses in the choice condition required longer than in the directed condition. These findings suggest that the extra overall processing cost of making a choice in Experiment 1 was likely due to participants having difficulty in recalling their chosen response type. Nonetheless, we observed a significant interaction between type of instruction and honesty of response and an increase in errors for truths in the choice condition, just as we did in Experiment 1. The response time difference between lying and telling the truth was smaller when participants chose their response than when they were directed to do so. In particular, participants were slower to respond with the truth when they chose the response compared to when they were directed to do so, but lying was much less affected by the choice manipulation. No explanation based on retrieval of the decision can be invoked because the visual prompt provided was identical for both conditions. The choice condition, however, provided slightly more time in terms of preparation. This is because the time between the participant making the choice and pressing the appropriate key would have to be added to the 1000 ms preparation time that is available in both choice and directed conditions. The fact that there is still a significant difference between time to lie and time to tell the truth means that this additional preparation time does not negate the key findings.

Neither of the decision making mechanisms that we discussed in Experiment 1 were borne out by the data. It is not the case that telling the truth is always the default option and that people have to choose to lie but not to tell the truth, otherwise we would have observed larger differences between truths and lies in the choice condition than the directed condition, nor is it the case that needing to choose a response is simply more difficult overall than being directed to respond. The decision mechanism involved in choosing whether to lie is therefore more complex than previously thought [34]. Our suggestion for how the decision mechanism functions is as follows. First, we assume that when people lie they must necessarily suppress the truthful response. This accounts for longer latencies for lies relative to truths in both choice and directed conditions. In addition, when people have to make an active decision of how to respond, the evaluation of these competing response possibilities is likely to invoke conflict monitoring processes. The conflict of choosing between a truth or lie response, compared to no such action being required in the directed condition, leads to overall longer response times for the choice condition. This evaluation of competing responses in authentic decisions is represented overtly when participants choose between a T or L response on the keyboard. Once individuals have considered these competing possibilities and made a response decision, the alternative, unused response will then require suppression. This suppression of the alternative response requires longer processing time for both lie and truth responses. Since liars are already suppressing the alternative response (the truth) on directed trials, this suppression only represents an additional process on choice trials for truth tellers, who now have to suppress a lie response.

It should be noted, however, that the findings of these two experiments relate specifically to questions where only one response alternative to the truth is available, such as yes-no questions. These findings have yet to be confirmed with questions involving more than one lie response option, although there is no reason to believe that the overall pattern of findings relating to the decision process would differ.

## Experiment 3

In Experiments 1 and 2 participants did not have a choice about which lie they told. When the square was red, for example, they had to lie with “blue,” and vice versa. The lie construction element was therefore minimal. Lying is often more complicated than this however, because liars have to construct a lie from a range of alternatives, as we discussed in the Introduction. Experiment 3 investigated which parts of the lie construction process contribute to longer response times.

We manipulated the range of lie and truth responses available to participants. In one condition, the square could be of one of two colors, as in Experiments 1 and 2. This is similar to yes-no questions, as in “Is your hair brown?” In the other condition the square could be one of three colors, similar to more open-ended questions, such as “What color is your hair?” The three-color trials therefore required a choice about which lie to use, whereas the two-color trials did not. All participants were directed about whether to lie, as in the directed conditions of Experiments 1 and 2. If the need to choose a lie contributes to the greater difficulty of lying, longer lie response times will be observed in the three-color lie condition than the two-color lie condition. Alternatively, longer response times might be observed in the three-color condition for both lie and truth responses.

### Method

#### Participants

Thirty-six Cardiff University students participated in this study in exchange for payment. Of these, 26 were female. Participants had a mean age of 21.83 (*SD*  =  3.60; *Range*  =  18–38) and spoke English as their first language.

#### Design

We used a 2 x 2 design with honesty of response (lie vs. truth) and number of response possibilities (two-color vs. three-color) as within-subjects factors. The dependent variable was response time. The paradigm consisted of two blocks of trials. The two-color block showed participants one of two colored squares and their lie response could only be the opposite color (hence one possible answer). The three-color block showed participants one of three colored squares and their lie response could be either of the other two colors (therefore a choice of two possible answers). The order of these blocks was counterbalanced across participants to minimise order effects. The color pair that participants were given in the two-color block (red/green, green/blue, blue/red) was also counterbalanced across participants so that all color combinations were present in all conditions. Participants took part in a practice block of 12 trials identical to the main trials. A total of 202 main trials were used in the paradigm: 100 in the two-color condition and 102 in the three-color condition.

#### Procedure

**Figure 5:**
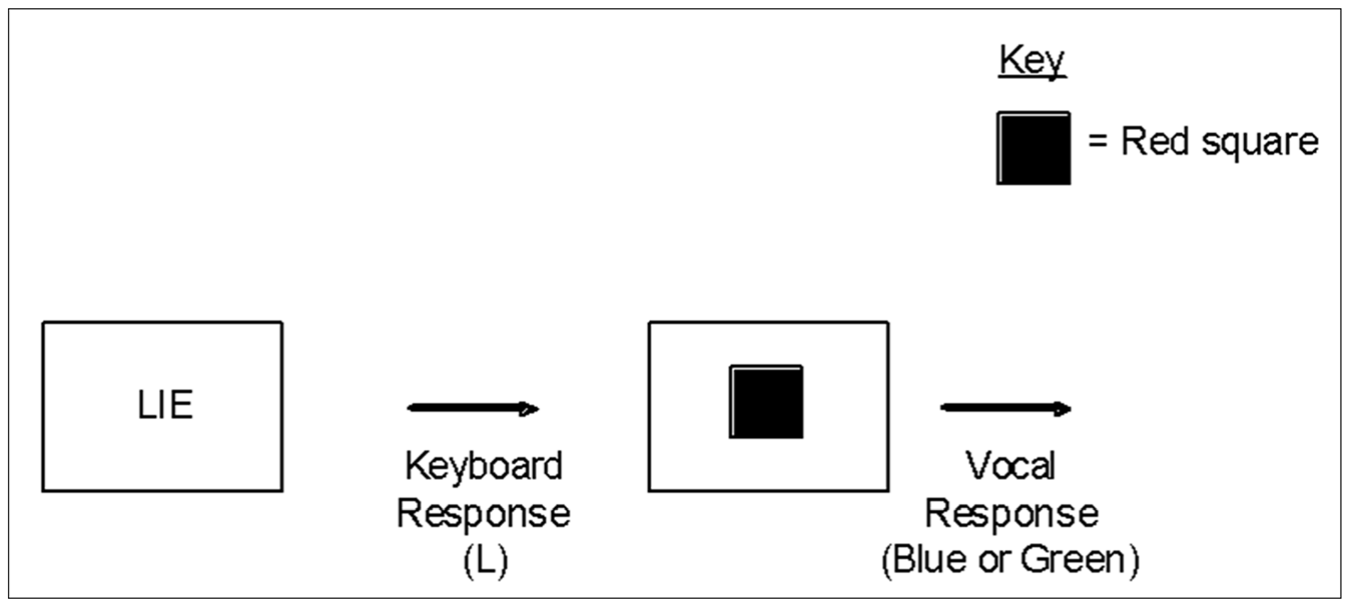
Example of a three-color lie trial from Experiment 3.

As in Experiment 1, the task involved the presentation of one of two words in the centre of the computer screen (LIE or TRUTH) and participants indicated that they understood by pressing the ‘T’ key when presented with the word ‘TRUTH’ and the ‘L’ key when presented with the word ‘LIE’. A colored square (blue, red or green) was then presented. Participants were required to lie or tell the truth about the color seen. Responses were recorded using a voice key. An example trial is shown in Figure 5.

### Results

There were 181 outliers (2.5%) in total and 62 of these were a result of microphone problems. No responses were less than 100 ms. These were removed from the analysis. Inaccurate responses (175) were also removed from the analysis. There were 38 (2.1%) inaccurate responses in the two-color lie condition and 50 (2.8%) in the two-color truth condition, X^2^(1)  =  1.7, *p* > 0.05. There were 51 (2.7%) inaccuracies in the three-color lie condition and 36 (2.0%) in the three-color truth condition, X^2^(1)  =  2.6, *p* > 0.05. Altogether, 356 out of 7,272 data points were removed from the analysis.

**Figure 6:**
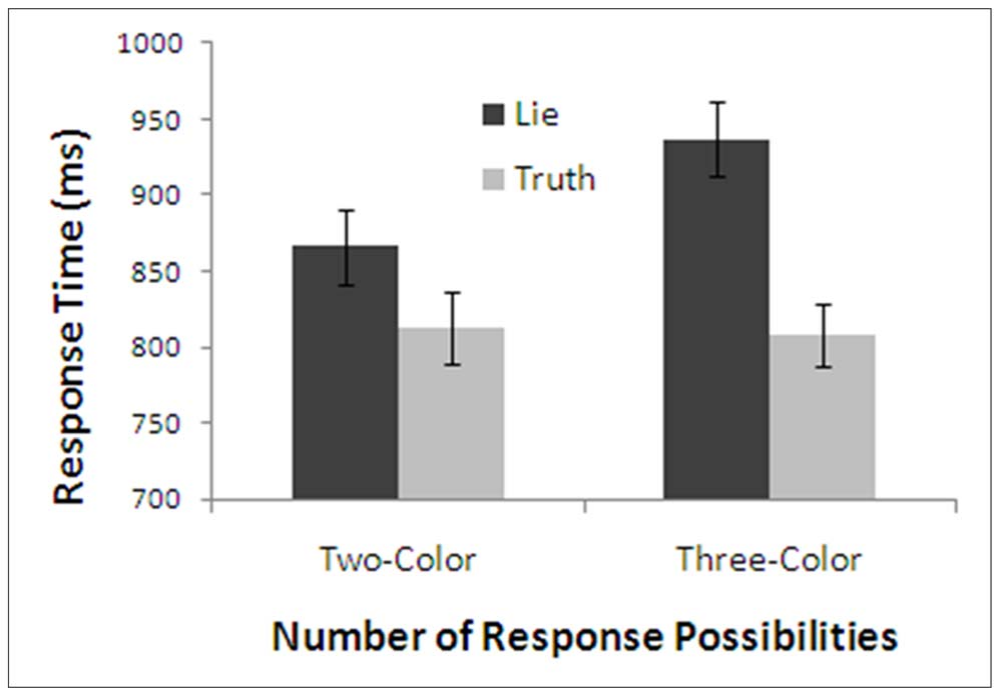
Response times of Experiment 3 as a function of number of response possibilities and honesty. Note: Error bars are standard error.

Mean response times for the four possible treatment conditions are presented in Figure 6. A repeated measures ANOVA was conducted with factors of honesty of response and number of response possibilities. Consistent with Experiment 2, telling a lie took longer than telling the truth, *F*(1,35)  =  139.79, *p* < .001, η^2^  =  .80. There was also a main effect of number of response possibilities, *F*(1,35)  =  4.11, *p* < .05, η^2^  =  .10 and a significant interaction, *F*(1,35)  =  31.78, *p* < .001, η^2^  =  .48, showing the lie-truth difference was significantly larger in the three-color condition than in the two-color condition. Simple main effects analysis revealed that the effect of honesty of response was significant in the two-color condition, *F*(1,35)  =  46.51, *p* < .001, η^2^  =  .57 and in the three-color condition, *F*(1,35)  =  112.02, *p* < .001, η^2^  =  .76. The interaction was driven by longer response times for lying to questions in the three-color condition compared to questions in the two-color condition, (Two-Color - Lie: *M*  =  866.16, *SD*  =  153.13; Three-Color - Lie: *M*  =  937.41, *SD*  =  153.07; *F*(1,35)  =  12.51, *p* < .001, η^2^  =  .26), and no effect of number of possible responses on truthful responding, (Two-Color - Truth: *M*  =  812.86, *SD*  =  141.86; Three-Color - Truth: *M*  =  807.94, *SD*  =  122.67; *F*(1,35)  =  0.11, *p*  =  .74, η^2^ < .01, *CI*  =  [–25, 35]).

In order to identify whether participants used one particular color more often than any other, we also examined which colors participants chose when they lied in the three color condition. Red was chosen 33% of the time, blue 35% of the time and green31% of the time. However, none of the colors were chosen more often than chance, *t*(35)*’*s < 1.40, *p’*s > .18.

### Discussion

In Experiment 3 we found that lying takes longer than telling the truth in both color conditions. More interestingly, we also found that there was a greater difference between lying and telling the truth in the three-color condition compared to the two-color condition. The interaction was driven by a significant increase in the time taken to lie to three-color compared with two-color questions and a nonsignificant difference in the time taken to tell the truth, consistent with the claim that lie construction is a costly process. Unlike other studies that have tested the difference between different question-types [31], our findings cannot be explained by differences in question content across conditions.

There are at least two explanations for why we observed a larger cost of lying in the three-color condition compared to the two-color condition. The first is that participants had to choose a lie in the three-color condition but not in the two-color condition (the lie was simply the one remaining option in the two-color condition). Having to make any kind of choice may have slowed participants down. The second is that participants could have been evaluating each of the possible lie responses in turn for their acceptability. Because there were twice as many possible lie responses in the three-color condition compared to the two-color condition, participants would have had to evaluate twice as many possibilities in the three-color condition than the two-color condition. There may be both a fixed cost of choosing and a cost to evaluating each alternative, or there could be one or other. In Experiment 4 we test whether participants evaluate each alternative.

## Experiment 4

If participants evaluate each of the possible lie responses in turn, expanding the range of possible lie options should continue to add time onto lie latencies. Conversely, if the cost we observed is a choice cost, expanding the range of options should not result in a proportional increase in lie latencies (there would be a single choice cost regardless of the number of possible lie responses). Experiment 4 tested these explanations by comparing trials with two possible lie responses (a *three-color* condition, as in Experiment 3) against trials with three possible lie responses (a *four-color* condition).

### Method

#### Participants

Thirty-two Cardiff University students participated in this study in exchange for course credit. Of these, 29 were female. Participants had a mean age of 18.94 (*SD*  =  0.95; *Range*  =  18–21) and spoke English as their first language.

#### Design

We used a 2 x 2 within-subjects design, with honesty of response (lie vs. truth) and number of response possibilities (three-color vs. four-color) as within-subjects factors. The dependent variable was response time. The paradigm consisted of two blocks of trials. The three-color block showed participants one of three colored squares and their lie response could be either of the other two colors (hence two possible answers). The four-color block showed participants one of four colored squares and their lie response could be any of the other three colors (hence three possible answers). The order of these blocks was counterbalanced across participants to prevent order effects. The colors that participants were given in the three-color block (red/green/blue, green/blue/purple, blue/purple/red, purple/red/green) were also counterbalanced across participants so that all color combinations were present in all conditions. Participants took part in a practice block of 12 trials identical to the main trials. A total of 202 main trials were used in the paradigm.

#### Procedure

The procedure was identical to that used in Experiment 3 except that participants saw one of four colored squares in the four-color condition.

### Results

There were 174 outliers (2.7%) in total. 78 of these were due to microphone problems. These were removed from the analysis. Inaccurate responses (260) were also removed from the analysis. No responses were less than 100 ms. There were 69 (4.3%) inaccurate responses in the three-color lie condition and 75 (4.7%) in the three-color truth condition, X^2^(1)  =  0.3, *p* > 0.05. There were 59 (3.7%) inaccuracies in the four-color lie condition and 57 (3.6%) in the four-color truth condition, X^2^(1)  =  0.1, *p* > 0.05. Altogether, 434 out of 6,464 data points were removed from the analysis.

**Figure 7:**
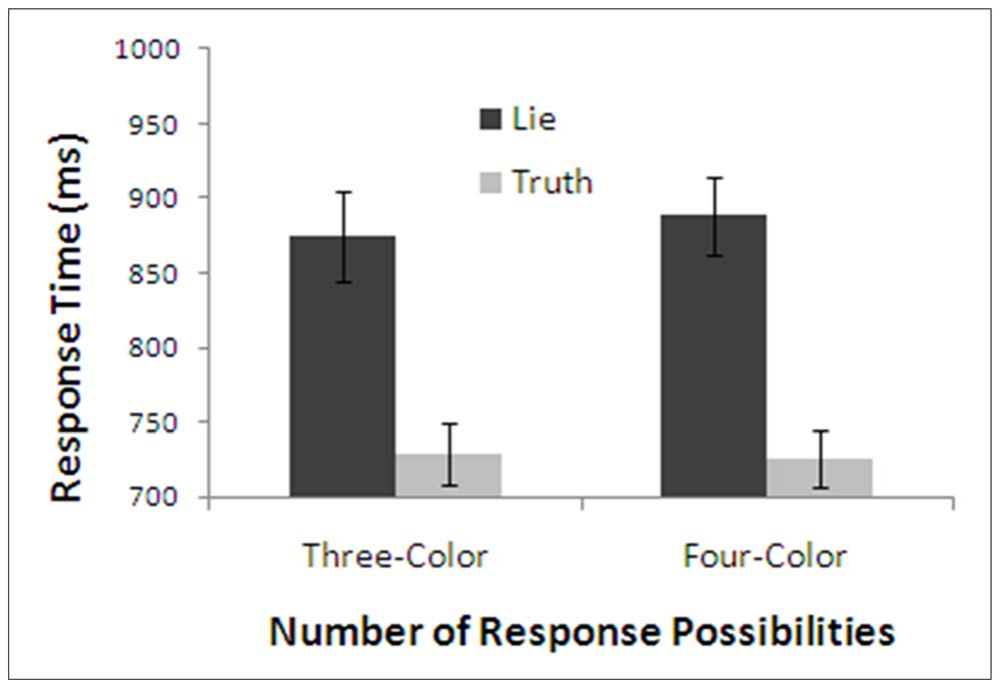
Response times of Experiment 4 as a function of number of response possibilities and honesty. Note: Error bars are standard error.

Mean response times for the four possible treatment combinations are presented in Figure 7. A repeated measures ANOVA was conducted with factors of honesty of response and number of response possibilities. This found a significant main effect of honesty of response with true responses being faster than lie responses, *F*(1,31)  =  117.06, *p* < .001, η^2^  =  .79. However, in contrast to the findings of Experiment 3, a further increase in the number of possible lie responses did not affect response times in either the truth, (Three-Color - Truth: *M*  =  728.96, *SD*  =  121.51; Four-Color - Truth: *M*  =  726.17, *SD*  =  106.90; *F*(1, 31)  =  0.04, *p*  =  .84, η^2^ < .01, *CI*  =  [-25, 30]), or lie conditions, (Three-Color - Lie: *M*  =  875.34, *SD*  =  171.42; Four-Color - Lie: *M*  =  888.39, *SD*  =  148.72; *F*(1, 31)  =  0.35, *p*  =  .56, η^2^ < 05, *CI*  =  [–58, 32]), nor was the interaction between number of response possibilities and honesty of response significant, *F*(1, 31)  =  0.57, *p*  =  .46, η^2^ < .02, showing that the lie-truth difference was not significantly larger in the four-color condition than in the three-color condition. A power analysis revealed that if the interaction was as large as we found in Experiment 2, i.e., η^2^  =  .26, we would have had a 99% chance of finding the effect.

As in Experiment 3, we investigated how participants chose their lie response. In the 3-color block, participants chose red 36% of the time, blue 31% of the time, green 31% of the time and purple 28% of the time. A one-sample t-test found that purple was used less than would be expected by chance, *t*(23)  =  2.53, *p* < .05, but that red, blue and green were not, *t*(23)*’*s < 1.70, *p’*s > .11. In the 4-color block, participants chose red 29% of the time, blue 20% of the time, green 27% of the time and purple 18% of the time. A one-sample t-test found that red was used more than chance, *t*(31)  =  2.28, *p* < .05, whereas blue, *t*(31)  =  3.18, *p* < .005 and purple, *t*(31)  =  3.58, *p* < .001 were used less than chance. The use of the green did not significantly differ from chance, *t*(31)  =  0.83, *p*  =  .41.

### Discussion

The results of Experiment 4 support previous findings of increased response times when individuals lie compared to when they tell the truth, regardless of the number of possible lie responses available. We also found that the number of possible lie responses did not significantly affect response times when individuals told the truth, consistent with the results of Experiment 3. Unlike Experiment 3, however, in this experiment no significant differences were demonstrated when individuals lied in the three-color compared to the four-color block and a power analysis indicated that we had a 99% chance of detecting an effect of the same size as that observed in Experiment 3. The processing time difference between questions with multiple response possibilities and those with only one response option is therefore likely to be due to the cost of choosing between lies in working memory, and not due to costs associated with evaluating each possible lie response in turn. We are not arguing that participants will never consider additional lie options in turn (or that lie times will never increase with options greater than three); rather, that the cost of having to choose *per se* will always be at least part of the extra cost of lying in multiple lie contexts.

It can be argued that individuals use a variety of strategies when generating lies in authentic settings, such as manipulating truthful information [38], and that our paradigm prevents this, and as such, prevents generalization to authentic settings. Indeed, our paradigm severely limits the available lie responses. However, three points should be considered here. Firstly, there are many situations that require individuals to complete the relatively simple task of choosing a lie response from a predetermined set of possibilities. For example, if asked the color of someone’s hair, individuals can choose between a predetermined set of acceptable hair colors in creating their lie response. Secondly, there are certain situations whereby lies are entirely false and do not involve any manipulation of the truth, such as denying recognising a well known acquaintance. Thirdly, it could be considered that using a different color as the lie response is to some extent an alteration of the truth, and as such, all lies involve a degree of alteration of truthful information, regardless of the specific context of the individual lie. Further considerations relating to lie selection, specifically the differing plausibility and acceptability of particular lies, are now addressed in Experiment 5.

## Experiment 5

In our previous experiments we showed that choosing between multiple lie responses increases response time. It should also be considered, however, that for the majority of lies some responses will be more plausible than others and the successful liar will need to consider this when selecting their response. The more plausible a response, the more likely that it will be chosen above other possibilities, since this increases the likelihood that a lie will be believed. In order to prevent implausible responses being used as a lie, like the truth, they become unacceptable answers to questions and must be suppressed alongside truthful information. What makes the task even more difficult is that a particular response is not necessarily implausible *per se* but depends on the question asked and the context (much like the truth). For example, “On the moon” would be a perfectly plausible (or truthful) answer to some questions, just not the location of the stolen money. Overall then, in any deceptive interaction there will be particular lies that cannot be used if the deception is to be successful. This discrimination of plausible and implausible lies can be considered a form of rule constraint, with limitations on the particular response that can be effectively used.

We are not aware of any evidence, however, that directly addresses the question of how implausible responses are discriminated from plausible responses, or how they are suppressed when people lie. One possibility is that plausibility computations are carried out in long term memory and that only plausible responses are transferred to working memory to be articulated. The ADCM assumes a similar process. An alternative, however, is that since lying is arguably an act that works against standard communicative principles [11], plausibility constraints may have to be implemented at a higher level than other language mechanisms. In order to override the use of truthful information when answering a question, lying may involve explicit, goal-oriented suppression of the default response. This may require distinct processes to be implemented in working memory. Experiment 5 was designed to test between these two accounts.

Participants engaged in a color naming task similar to Experiments 3 and 4. The difference was that in Experiment 5 we introduced constraints on which lies (colors) participants could use. Specifically, we told participants that they would have to name squares of three different colors, red, green, and blue either truthfully or untruthfully, but that they were not allowed to lie with one of the colors (red, say). We therefore had lie and truth trials. In the lie trials they would have to say whatever color was presented whether it be green, blue or red. The lie trials were broken down depending on the plausibility constraint. When the colored square was the disallowed lie color (red), participants had the choice of two lie possibilities (blue and green). We refer to these as lie *control* trials because the lie possibilities were the same as if no constraint was introduced. When the square was one of the allowed lie colors (green, say), participants could not say the prohibited lie color (red) and hence had to choose the other lie color (blue). These were lie *constraint* trials.

If plausibility constraints are implemented in long term memory, only allowable responses would be transferred into working memory. In the lie control trials, this would mean two potential lie responses, that is, green and blue, but in the lie constraint trials, only one possible response would be available, i.e., green (or blue). From Experiment 3 we know that lying with two possible responses is more difficult than lying with only one possible response, hence RTs in the lie control trials should be slower than those in the lie constraint trials. Alternatively, if plausibility constraints are implemented in working memory, participants would have two lie responses in working memory in both conditions. They would then have to explicitly suppress the disallowed lie response in the lie constraint condition, which should take additional time, as it did when participants suppressed the truthful response throughout Experiments 1–4. RTs to the lie constraint condition should therefore be higher than in the lie control condition.

### Method

#### Participants

Thirty undergraduate psychology students volunteered for this study in exchange for course credit. Of these, 29 were female. Participants had a mean age of 20 (*SD*  =  3.2; *Range*  =  18–33) and spoke English as their first language.

#### Design

A 2x2 within-subjects design was used, with honesty of response (truth vs. lie) and plausibility (constraint vs. control) as within-subjects factors. The dependent variable was response time measured in milliseconds (ms). A total of 408 trials were included in the main experimental task, with 68 from the lie control condition, 68 from the truth control condition, 136 from the lie constraint condition and 136 from the truth constraint condition. The order of trials was randomised for each participant.

#### Procedure

A similar paradigm was used to Experiments 3 and 4, with the presentation of either the word TRUTH or LIE in the centre of the computer screen. Once again, participants pressed the ‘T’ key when presented with the word TRUTH and the ‘L’ key when presented with the word ‘LIE’. This was followed by the presentation of either a blue, green or red square. As before, participants then had to say either the true color of the square or lie about the color of the square by claiming that it was a different color. Prior to the main trials, participants completed a short practice block containing 4 trials.

In contrast with our previous experiments, participants were instructed that they could only use two of the presented colors as their lie response and could not use the third color as a lie answer (e.g., participants could use green red or blue but not red). The particular color (red, blue or green) that participants were instructed against using as a lie was counterbalanced across participants.

### Results

There were 264 outliers (2.2%) in total. 256 of these were due to microphone problems. These were removed from the analysis. No responses were less than 100 ms. Inaccurate responses (363) were also removed from the analysis. Overall, there were 55 (2.7%) inaccurate responses when participants lied in the control condition and 53 (2.6%) when participants told the truth in the control condition, X^2^(1)  =  0.1, *p* > 0.05. There were 162 (4.0%) when participants lied in the constraint condition, and 93 (2.3%) when participants told the truth in the constraint condition, X^2^(1)  =  19.2, *p* > 0.05. In total, 627 out of 11,970 data points were removed from the analysis.

**Figure 8:**
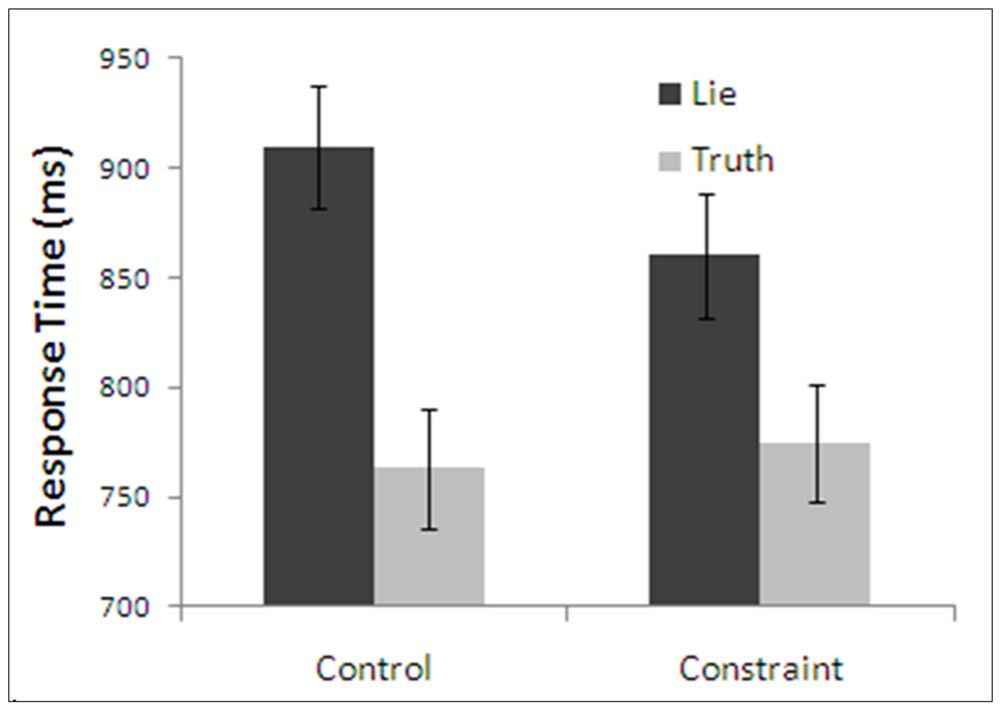
Response times of Experiment 5 as a function of truthful color and honesty. Note: Error bars are standard error.

A repeated measures ANOVA was conducted with honesty (truth vs. lie) and plausibility (constraint vs. control) as within-subjects factors. A main effect of honesty was demonstrated, *F*(1,29)  = 145.52, *p* < .001, η^2^  =  .83, such that lie response times were significantly longer than truth response times, for both control and constraint trials. In addition, a main effect of plausibility was demonstrated, *F*(1,29)  = 14.89, *p* < .005, η^2^  =  .34 and a significant interaction between honesty and plausibility, *F*(1,29)  =  23.27, *p* < .001, η^2^  =  .44, such that the lie-truth difference was significantly larger in the constraint condition than in the control condition. This interaction was due to significantly longer response times when participants lied in the control condition compared to the constraint condition (Lie - Control: *M*  =  909.56, *SD*  =  175.51; Lie - Constraint: *M*  =  860.16, *SD*  =  151.06; *F*(1,29)  = 40.48, *p* < .001, η^2^  =  .58). This finding is evidence in favour of constraints being applied in long-term memory. Little difference was shown between the two conditions when individuals told the truth (Truth - Control: *M*  =  762.73, *SD*  =  148.29; Truth - Constraint: *M*  =  774.53, *SD*  =  156.15; *F*(1,29)  = 2.06, *p*  =  .162, η^2^  =  .07). Mean response times for the four possible treatment combinations are shown in Figure 8.

### Discussion

The main effect of honesty of response shown in our previous experiments was also demonstrated in Experiment 5, with lying taking longer than telling the truth in both the constraint and control conditions. Two main predictions were considered regarding the choice between lie possibilities in relation to response plausibility. These focused on whether implausible lies entered working memory and were considered in the decision process, or whether such responses were inhibited prior to this in long-term memory systems. Our findings support the latter hypothesis because there were significantly longer lie responses in lie control trials compared to lie constraint trials. If both implausible and plausible lies were transferred to, and active in working memory, then a choice would be required between them (as seen in Experiment 3). This would result in little response time difference between the lie control and lie constraint conditions, since a choice would be required between two possible responses in both conditions. Our findings suggest instead that the implausible lie response is inhibited prior to this decision process, so a decision between the two possibilities is not required (since only one color can be plausibly used). This supports the suggestion (consistent with the ADCM) that implausible lies are inhibited in long-term memory and only plausible lies enter working memory systems.

## General Discussion

The aim of the current study was to investigate the cognitive processes that occur when people lie. Telling a lie typically takes longer than telling the truth and we were interested in understanding why. We organised our experiments around three potential contributing factors: suppressing a truthful response; the decision to lie; and the construction of a lie. We now summarize our results and describe their implications with respect to these factors.

### Suppression of the truthful response

In all of our experiments in which participants were instructed to lie, lying response times were longer than truthful response times. More interestingly, we observed this result under conditions in which many of the factors that are usually considered to slow down lying were absent. In particular, participants did not need to construct a plausible lie (in Experiments 1 and 2 only one possible lie response was available) nor did they need time to decide to lie (Experiments 3, 4 and 5 removed the decision process completely). According to models such as the ADCM Revised [34], the only process left to explain longer lie response times is that the truthful response needs to be suppressed. Our experiments therefore provide direct evidence that suppression of the truthful response is a contributing factor to longer lie response times.

While we agree that suppression is part of the explanation, it is important to outline the different mechanisms by which suppression might lead to slower response times. One possibility is that lying is a multi-stage, serial processing mechanism in which the truthful response is retrieved and enters into working memory first, it is then rejected (because a lie is needed), and then a lie response retrieved. Telling the truth, in contrast, is only a single-stage processing mechanism, in which the truthful response is retrieved and enters into working memory. Under this account, the difference in response times between lies and truths is due to having to retrieve two responses in the lie condition (the lie and the truth) and only one in the truth condition (the truth). An alternative but similar proposal is that lying involves rejecting a response, whereas telling the truth does not. Perhaps rejection is a conscious process that takes time.

A more distinct alternative is that the processes that underlie suppression of the truth occur in parallel, and in long-term memory, not in serial, short term memory. Assuming that response time is determined by variation in activation levels across the response possibilities (with large differences in activation levels being associated with short response times), reducing the activation of the truthful response might reduce overall variation in activation levels. This would make it more difficult to generate a response when lying than when telling the truth because it would be more difficult to select one response over the others. While this might explain why lying takes longer than telling the truth on some occasions, it is unlikely to be a general explanation. First, recent brain imaging research has found increased activation of brain areas associated with working memory when individuals lie [22]. The extra cost of lying cannot therefore be restricted to long-term memory under all circumstances. Second, lying involves deliberately choosing not to say the truth [46]. Now, since working memory is typically associated with conscious awareness [47], lying should involve truthful responses entering working memory (and being suppressed in working memory).

The two types of suppression that we have identified may both be correct but apply under different circumstances. Serial suppression in working memory is likely to be the more standard, day-to-day type of suppression in which a speaker lies to an unexpected question on a single occasion. However, if a speaker has to lie on multiple occasions to the same question, or they are in a situation in which lying is likely to be common and expected, they may be able to suppress truthful answers in long-term memory, almost “forgetting” the truth because the lie response has been so frequently associated with a given question.

### The decision to lie

Experiments 1 and 2 tested the role of the decision process by comparing response times in trials in which participants chose to lie with trials in which they were directed to lie. While we found effects of deciding to lie in both of our experiments, we discovered that there was a much greater cost to deciding to tell the truth than deciding to lie, relative to the cost of being directed in the response. Thus, although it has been suggested that the decision contribution to elevated lie response times is at least partially determined by the difficulty in lying [34], our data show that this process also occurs for decisions related to truthful responses. Our general view is therefore that there is no cost of deciding to lie *per se* but there is a cost to choosing to depart from the norm for that context. Most of the time when people lie they will be departing from a truth-telling context, which is likely to incur a cost, but in some contexts, e.g., interrogation situations, or playing poker, delays may be experienced when the decision is taken to tell the truth.

One caveat to our conclusion is that when people choose to lie they often do so on the basis of the question that they are asked, whereas in our experiments the choice was internally driven. For example, a person may choose to lie to questions about the whereabouts of a suspect but not about their own activities. Evaluating the content of the question is a component of the decision process which is not included in our task. It could therefore be that the evaluation component of the decision process contributes to elevated lie latencies. However, we feel that this cost is also caused by a departure from the normal communicative stance. This is because if the person would normally tell the truth, the question needs to be evaluated in order to decide to lie, but if the person expects to lie, the question needs to be evaluated in order to decide whether to tell the truth. Thus, the departure from the norm is the causal factor, not the decision to lie.

Second, we observed longer response times when participants told the truth in the choice condition compared to the directed condition. This occurred across both experiments and therefore was not related to differential visual availability of the response type across conditions. As a consequence of this effect, the difference between lying and telling the truth was greatly diminished in the choice conditions (to the extent that we did not observe a significant difference in Experiment 1). What is different about choosing to lie compared to being directed to lie? One hypothesis is that choosing to lie means considering lie and truthful responses. For example, when deciding whether to lie to a red square, the responses “blue” (the lie) and “red” (the truth) become activated. Consequently, in our study, there was a small (or nonexistent) response time difference between truthful and lie responses in the choice condition because both responses were highly activated under both response conditions. In contrast, being directed to tell the truth means that only the truthful response becomes activated (there is no need to consider and suppress the lie response), but being directed to lie means that the truth and the lie response become activated (the truth is always activated). In other words, both response types were activated in the choice-lie, directed-lie, and choice-truth conditions, but only the truth was activated in the directed-truth condition.

Finally, these results should be considered in relation to practical situations. In almost all lie detection work participants are directed to lie or tell the truth rather than choosing to do so whereas when people lie in everyday situations, they choose to lie rather than being directed. Our experiments show that the difference between lying and telling the truth is much smaller when participants are given a choice. This should certainly be considered in further work targeted at more practical settings, since such lies may therefore be less detectable when using automated lie detection techniques.

### The construction of a lie

There is a strong intuition that lying takes longer than telling the truth because lies need to be constructed whereas truths do not. Yet, the evidence we reviewed in the Introduction was inconclusive about why, or even whether, this was the case. Our experiments make two novel contributions to understanding the construction component of lying.

First, having to make a choice about which lie to use from many, arbitrary possibilities is difficult. Experiments 3 and 4 demonstrated that when participants had to choose a lie they were slow at responding, but, crucially, the same range of response options did not slow truthful responses. Even after hundreds of trials, and with only two choices, participants experienced difficulty in making an arbitrary choice when they were forced to lie. It seems that part of what makes lying difficult is resolving all of the inconsequential decisions that are needed in order to construct a story. When telling the truth, the “decisions” are determined by fact, or by memory, and are therefore relatively resource free.

Second, and somewhat conversely, when there is a clear preference about which lie is the most appropriate, lying is relatively easy. In Experiment 5 we found that when participants were prevented from using one lie response out of two (but were required to use both responses when stating the truth), participants behaved as if there was only one possible lie available. Rejection of the implausible lie occurred in long term memory, as if no choice between lies was necessary. One caveat to this result is that our effects were obtained over many trials with the same plausibility constraint applied on each occasion. It may be the case that making plausibility assessments in unrehearsed lie situations is much more difficult. We leave this investigation to future research, however.

Our results on lie construction additionally make one suggestion that contrasts with previous claims that yes/no questions provide better indicators of deceit than open-ended questions [1], [31], [34]. These claims are based on findings of greater response time differences between lies and truths when participants lied to yes/no compared to open-ended questions. In contrast, we found a greater difference for questions with more than one possible lie response. We suggest that different patterns arose because different methodologies were used across studies. In our experiments, participants answered the same type of question in both conditions and the truthful answer was equally accessible across conditions. In the above cited papers, however, different types of questions were asked across conditions and the truthful answer could have been more difficult to retrieve in the open-ended questions (hence truthful response times were longer in the open-ended condition). While we agree that the difficulty of retrieving truthful information contributes to the response time difference between lies and truths, we feel that this issue is orthogonal to the issue of yes/no vs. open-ended questioning. The results of our experiments on lie construction suggest that an interviewee may need more time to lie to an open-ended question than to a yes/no question, *ceteris paribus*, because they need to choose which lie to use in the open-ended case but not in the yes/no case. Before any firm conclusions can be drawn regarding the effect of question type on the optimisation of deception detection, however, the likely accessibility of truthful information and the situational context should be further examined.

### Limitations and future directions

The paradigm that we used appears quite different to the usual methods of investigating how people lie [10], [48]. For example, participants were not asked to lie about personal information, nor was there an interlocutor present asking questions. Further, there was no incentive to lie, which should have meant that there were no stress effects. We argued in the introduction that the method we employed is a powerful technique without which we would not have been able to address the detailed processing questions discussed above. It is important, however, to consider the relationship between our task and lying outside of the laboratory.

Similar to many cognitive experiments [15], [21], [29], [49], our paradigm did not require participants to engage in the direct deception of another individual. They were producing verbal responses recorded by a computer, and there was no human “addressee” to fool. While this procedure means that participants may have felt that the task was different to lying in everyday life, they were performing operations that must necessarily be present in even the most simple of lies independently of both the intention and motivation to deceive. What is important is that participants in our study intentionally and knowingly produced falsehoods. While there are situations in which a person can knowingly produce falsehoods without lying (e.g., when both parties are aware of the falsehood) there are very few situations when lies are produced without falsehoods [50]. Clearly, however, it is possible that the effects found in our experiments may interact or be overshadowed by the affective components of lying, such as guilt, stress or negative emotions in general. Future studies may be able to test these interactions by, for example, inducing negative moods in participants in the laboratory [51], [52].

Atypically for research in deception, participants in the current study had to lie when a representation of the truth was in front of them. For example, participants had to lie, “red” when the truth, a yellow square, was present on the screen (compare this with a study in which participants are asked to lie about having performed an everyday act [53]). One likely effect of having the visual stimulus on the screen would be to make it more difficult to suppress the truthful response when lying. This design, therefore, maximised the suppression effect so we could manipulate particular components of the lie process. Despite the likelihood of larger effects, however, there is no reason why the overall difficulty should have interacted with the difference between choosing to lie and being directed to lie (Experiments 1 and 2) or the difference between one and two or three plausible and implausible lie possibilities (Experiments 3, 4 and 5). Both lying about a visual stimulus and lying about the content of memory involve suppression of the truthful response and the experiments reported here investigated this suppression. Furthermore, participants were not being presented with the color name, i.e., a possible response, only a colored square. This meant that the truthful response still needed to be recalled from memory, just as if we had asked them what they were up to the night before last.

Lastly, we acknowledge that only a single cue to deception was used as a measure of cognitive load. Although response times are a well regarded measure of cognitive processing, other researchers have recommended the use of multiple cues to detect deceit [54], including blink rate [55] and body movements [56], and this should be considered in practical lie detection settings.

## Conclusion

Despite the wealth of research investigating lying in general, such as lie detection [37], the social psychology of lying [3], [4] and the linguistics and philosophy of lying [50], very little work has been conducted on how we lie. Our study has tried to address the imbalance by investigating why people take longer to lie than to tell the truth. We come to three conclusions. First, lying involves suppressing truthful information and suppressing or rejecting a default response will increase response time. Second, there can be costs associated with choosing to tell the truth, just as there can be with choosing to lie. We therefore maintain that the decision to depart from the normal type of communication can be costly, and while this will often be a cost associated with a decision to lie, it is not an obligatory component of lying. Lastly, lying often requires more choice in generating a response than telling the truth. There is typically only one truth but there are many possible lie options. Making a choice about which lie to use is a difficult job and contributes to the longer time needed to tell a lie.

## References

[1] Walczyk JJ, Roper KS, Seemann E, Humphrey AM (2003) Cognitive mechanisms underlying lying to questions: Response time as a cue to deception. Applied Cognitive Psychology 17: 755–774.

[2] Serota KB, Levine TR, Boster FJ (2010) The prevalence of lying in America: Three studies of self-reported lies. Human Communication Research 36: 2–25.

[3] Cole T (2001) Lying to the one you love: The use of deception in romantic relationships. Journal of Social and Personal Relationships 18: 107–129.

[4] DePaulo BM, Kashy DA (1998) Everyday lies in close and casual relationships. Journal of Personality and Social Psychology 74: 63–79. 9457776 10.1037//0022-3514.74.1.63

[5] Seiter JS, Bruschke J, Bai C (2002) The acceptability of deception as a function of perceivers' culture, deceiver's intention, and deceiver-deceived relationship. Western Journal of Communication 66: 158–180.

[6] Appelbaum PS (2007) The new lie detectors: Neuroscience, deception, and the courts. Psychiatric Services 58: 460–462. 17412845 10.1176/ps.2007.58.4.460

[7] Inbau FE, Reid JE, Buckley JP, Jayne BC (2001) Criminal interrogation and confessions, fourth edition. Gaithersburg, Maryland: Aspen.

[8] Porter S, ten Brinke L (2009) Dangerous decisions: A theoretical framework for understanding how judges assess credibility in the courtroom. Legal and Criminolgical Psychology 14: 119–134.

[9] Raskin DC, Esplin PW (1991) Assessment of children's statements of sexual abuse. In: Doris J, editor. The suggestibility of children's statements recollections. Washington D C: American Psychological Association. pp. 153-165.

[10] Vrij A, Mann S, Fisher RP, Leal S, Milne R, et al (2008) Increasing cognitive load to facilitate lie detection: The benefit of recalling an event in reverse order. Law and Human Behavior 32: 253–265. 17694424 10.1007/s10979-007-9103-y

[11] Grice HP (1989) Studies in the way of words. Cambridge, Massachusetts: Harvard University Press.

[12] Ganis G, Rosenfeld JP, Meixner J, Kievit RA, Schendan HE (2011) Lying in the scanner: covert countermeasures disrupt deception detection by functional magnetic resonance imaging. Neuroimage 55: 312–319. 21111834 10.1016/j.neuroimage.2010.11.025

[13] Holden RR (1998) Detecting fakers on a personnel test: Response latencies versus a standard validity scale. Journal of Social Behavior and Personality 13: 387–398.

[14] Vendemia JMC, Buzan RF, Green EP (2005) Practice effects, workload, and reaction time in deception. American Journal of Psychology 118: 413–428. 16255127

[15] Vendemia JMC, Buzan RF, Simon-Dack SL (2005) Reaction time of motor responses in two-stimulus paradigms involving deception and congruity with varying levels of difficulty. Behavioural Neurology 16: 25–36. 16082077 10.1155/2005/804026 PMC5478843

[16] Verschuere B, Crombez G, Degrootte T, Rosseel Y (2010) Detecting concealed information with reaction times: Validity and comparison with the polygraph. Applied Cognitive Psychology 24: 991–1002.

[17] Zuckerman M, DePaulo BM, Rosenthal R (1981) Verbal and nonverbal communication of deception. In: Berkowitz L, editor. Advances in experimental social psychology. New York: Academic Press. pp. 1-57.

[18] Abe N, Okuda J, Suzuki M, Sasaki H, Matsuda T, et al (2008) Neural correlates of true memory, false memory, and deception. Cerebral Cortex 18: 2811–2819. 18372290 10.1093/cercor/bhn037 PMC2583150

[19] Lee TMC, Liu HL, Chan CCH, Ng YB, Fox PT, et al (2005) Neural correlates of feigned memory impairment. NeuroImage 28: 305–313. 16165373 10.1016/j.neuroimage.2005.06.051

[20] Spence SA, Farrow TF, Herford AE, Wilkinson ID, Zheng Y, et al (2001) Behavioral and functional anatomical correlates of deception in humans. Neuroreport 12: 2849–2853. 11588589 10.1097/00001756-200109170-00019

[21] Spence SA, Kaylor-Hughes C, Farrow TFD, Wilkinson ID (2008) Speaking of secrets and lies: The contribution of ventrolateral prefrontal cortex to vocal deception. NeuroImage 40: 1411–1418. 18308586 10.1016/j.neuroimage.2008.01.035

[22] Christ SE, Van Essen DC, Watson JM, Brubaker LE, McDermott KB (2009) The contributions of prefrontal cortex and executive control to deception: Evidence from activation likelihood estimate meta-analyses. Cerebral Cortex 19: 1557–1566. 18980948 10.1093/cercor/bhn189 PMC2693617

[23] Lykken DT (1959) The GSR in the detection of guilt. Journal of Applied Psychology 43: 385–388.

[24] Lui M, Rosenfeld JP (2008) Detection of deception about multiple, concealed, mock crime items, based on a spatial-temporal analysis of ERP amplitude and scalp distribution. Psychophysiology 45: 721–730. 18665865 10.1111/j.1469-8986.2008.00683.x

[25] Rosenfeld JP, Shue E, Singer E (2007) Single versus multiple probe blocks of P300-based concealed information tests for self-referring versus incidentally obtained information. Biological Psychology 74: 396–404. 17126984 10.1016/j.biopsycho.2006.10.002

[26] Verschuere B, Rosenfeld JP, Winograd M, Labkovsky E, Wiersema JR (2008) The role of deception in the P300-based concealed information test. International Journal of Psychophysiology 69: S149.

[27] Seymour TL, Fraynt BR (2009) Time and encoding effects in the concealed knowledge test. Applied Psychophysiology and Biofeedback 34: 177–187. 19536648 10.1007/s10484-009-9092-3 PMC2727398

[28] Verschuere B, De Houwer J (2011) Detecting concealed information in less than a second: Response latency-based measures. In: Vershuere B, Ben-Shakhar G, Meijer E, editors. Memory detection: theory and application of the concealed information test. Cambridge, UK: Cambridge University Press. pp. 46-62.

[29] Johnson Jr R, Barnhardt J, Zhu J (2004) The contribution of executive processes to deceptive responding. Neuropsychologia 42: 878–901. 14998703 10.1016/j.neuropsychologia.2003.12.005

[30] Vendemia JMC, Schillaci MJ, Buzan RF, Green EP, Meek SW (2009) Alternate technologies for the detection of deception. In: Wilcox DT, editor. The use of the polygraph in assessing, treating and supervising sex offenders. West Sussex, UK: Wiley-Blackwell. pp. 267-296.

[31] Walczyk JJ, Schwartz JP, Clifton R, Adams B, Wei M, et al (2005) Lying person-to-person about life events: A cognitive framework for lie detection. Personnel Psychology 58: 141–170.

[32] Kintsch W (1998) Comprehension: A paradigm for cognition. Cambridge, England: Cambridge University Press.

[33] Ericsson KA, Kintsch W (1995) Long-term working memory. Psychological Review 102: 211–245. 7740089 10.1037/0033-295x.102.2.211

[34] Walczyk JJ, Mahoney KT, Doverspike D, Griffith-Ross DA (2009) Cognitive lie detection: Response time and consistency of answers as cues to deception. Journal of Business and Psychology 24: 33–49.

[35] Seymour TL, Seifert CM, Shafto MG, Mossman AL (2000) Using response time measures to assess 'guilty knowledge.'. Journal of Applied Psychology 85: 30–37. 10740954 10.1037/0021-9010.85.1.30

[36] Boaz TL, Perry NW, Raney G, Fischler IS, Schuman D (1991) Detection of guilty knowledge with event-related potentials. Journal of Applied Psychology 76: 788–795.

[37] Vrij A, Mann S, Kristen S, Fisher R (2007) Cues to deception and ability to detect lies as a function of police interview styles. Law and Human Behavior 31: 499–518. 17211691 10.1007/s10979-006-9066-4

[38] DePaulo BM, Lindsay JJ, Malone BE, Muhlenbruck L, Charlton K, et al (2003) Cues to deception. Psychological Bulletin 129: 74–118. 12555795 10.1037/0033-2909.129.1.74

[39] Granhag PA, Stromwall LA (2002) Repeated interrogations: Verbal and nonverbal cues to deception. Applied Cognitive Psychology 16: 243–257.

[40] Porter S, Doucette NL, Woodworth M, Earle J, MacNeil B (2008) Halfe the world knows not how the other halfe lies: Investigation of verbal and non-verbal signs of deception exhibited by criminal offenders and non-offenders. Legal and Criminological Psychology 13: 27–38.

[41] Ben-Shakhar G, Elaad E (2002) The guilty knowledge test (GKT) as an application of psychophysiology: Future prospects and obstacles. In: Kleiner M, editor. Handbook of polygraph testing. San Diego, CA: Academic Press. pp. 87-102.

[42] Ekman P, Friesen W (1974) Detecting deception from body or face. Journal of Personality and Social Psychology 29: 288–298.

[43] Bond GD, Lee AY (2005) Language of lies in prison: linguistic classification of prisoners' truthful and deceptive natural language. Applied Cognitive Psychology 19: 313–329.

[44] DePaulo BM, Kirkendol SE (1989) The motivational impairment effect in the communication of deception. In: Yuille JC, editor. Credibility assessment. Dordrecht, the Netherlands: Kluwer. pp. 51-70.

[45] Carrion RE, Keenan JP, Sebanz N (2010) A truth that's told with bad intent: An ERP study of deception. Cognition 114: 105–110. 19836013 10.1016/j.cognition.2009.05.014

[46] Spence SA, Hunter MD, Farrow TFD, Green RD, Leung DH, et al (2004) A cognitive neurobiological account of deception: Evidence from functional neuroimaging. Philosophical Transactions of the Royal Society of London 359: 1755–1762. 15590616 10.1098/rstb.2004.1555 PMC1693447

[47] Dehaene S, Naccache L (2001) Towards a cognitive neuroscience of consciousness: basic evidence and a workspace framework. Cognition 79: 1–37. 11164022 10.1016/s0010-0277(00)00123-2

[48] Abe N, Suzuki M, Mori E, Itoh M, Fujii T (2007) Deceiving others: Distinct neural responses of the prefrontal cortex and amygdala in simple fabrication and deception with social interactions. Journal of Cognitive Neuroscience 19: 287–295. 17280517 10.1162/jocn.2007.19.2.287

[49] Mameli F, Mrakic-Sposta S, Vergari M, Fumagalli M, Macis M, et al (2010) Dorsolateral prefrontal cortex specifically processes general - but not personal - knowledge deception: Multiple brain networks for lying. Behavioural Brain Research 211: 164–168. 20307584 10.1016/j.bbr.2010.03.024

[50] Meibauer J (2005) Lying and falsely implicating. Journal of Pragmatics 37: 1373–1399.

[51] Kirschbaum C, Pirke KM, Hellhammer DH (1993) The Trier Social Stress Test: A tool for investigating psychobiological stress responses in a laboratory setting. Neuropsychobiology 28: 76–81. 8255414 10.1159/000119004

[52] Philippot P (1993) Inducing and assessing differentiated emotion-feeling states in the laboratory. Cognition & Emotion 7: 171–193. 27102736 10.1080/02699939308409183

[53] Fullam RS, McKie S, Dolan MC (2009) Psychopathic traits and deception: A functional magnetic resonance imaging study. The British Journal of Psychiatry 194: 229–235. 19252152 10.1192/bjp.bp.108.053199

[54] Vrij A, Edward K, Roberts KP, Bull R (2000) Detecting deceit via analysis of verbal and nonverbal behavior. Journal of Nonverbal Behavior 24: 239–263.

[55] Fukuda K (2001) Eye blinks: New indices for the detection of deception. International Journal of Psychophysiology 40: 239–245. 11228351 10.1016/s0167-8760(00)00192-6

[56] Duran ND, Dale R, McNamara DS (2010) The action dynamics of overcoming the truth. Psychonomic Bulletin & Review 17: 486–491. 20702866 10.3758/PBR.17.4.486

